# Structure and gating of the nuclear pore complex

**DOI:** 10.1038/ncomms8532

**Published:** 2015-06-26

**Authors:** Matthias Eibauer, Mauro Pellanda, Yagmur Turgay, Anna Dubrovsky, Annik Wild, Ohad Medalia

**Affiliations:** 1 Department of Biochemistry, University of Zurich, Winterthurerstrasse 190, Zurich 8057, Switzerland; 2 Department of Life Sciences and the National Institute for Biotechnology in the Negev, Ben-Gurion University, Beer-Sheva 84105, Israel

## Abstract

Nuclear pore complexes (NPCs) perforate the nuclear envelope and allow the exchange of macromolecules between the nucleus and the cytoplasm. To acquire a deeper understanding of this transport mechanism, we analyse the structure of the NPC scaffold and permeability barrier, by reconstructing the *Xenopus laevis* oocyte NPC from native nuclear envelopes up to 20 Å resolution by cryo-electron tomography in conjunction with subtomogram averaging. In addition to resolving individual protein domains of the NPC constituents, we propose a model for the architecture of the molecular gate at its central channel. Furthermore, we compare and contrast this native NPC structure to one that exhibits reduced transport activity and unveil the spatial properties of the NPC gate.

## Abstract (web summary)

Nuclear pore complexes (NPCs) are large macromolecular assemblies that mediate the exchange of molecules between the nucleus and cytoplasm. Here the authors present a ∼20 Å cryo-EM structure of the *X. laevis* NPC in different states of transport to propose a model for the architecture of the NPC's molecular gate within its central channel.

## Graphical abstract

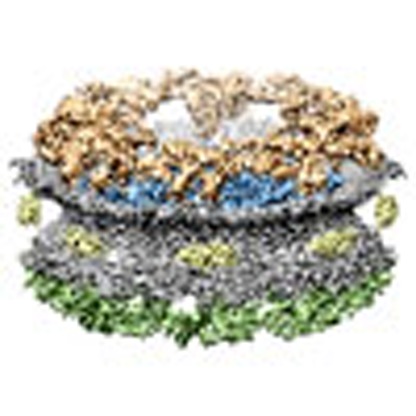

Nuclear pore complexes (NPCs) mediate the macromolecular exchange between the nucleoplasm and cytoplasm^1^,^2^. These supramolecular assemblies, 60–125 MDa in molecular weight^3^,^4^, constitute the sole gateway through the nuclear envelope (NE). They permit passive diffusion^5^ of ions and small molecules with a radius of ≲2.6 nm, while large cargos are actively transported by a signal-mediated mechanism using specific receptors^6^. Driven by the RanGTP/GDP cycle^7^, cargo–receptor complexes of up to ∼39 nm can pass the permeability barrier^8^. Electron microscopy studies^9^,^10^,^11^,^12^,^13^,^14^ revealed the three ring moieties of the NPC scaffold: the nucleoplasmic ring (NPR), the cytoplasmic ring (CPR) and the spoke ring (SR). The SR is sandwiched between the NPR and the CPR and harbours the central channel. Along the nucleocytoplasmic axis of transport the complex possesses an eightfold rotational symmetry^9^.

At the molecular level, NPCs are composed of about 30 different proteins^4^ termed nucleoporins (Nups). Some Nups assemble together into stable complexes. Nup107 is one such complex and it comprises of 10 Nups (Nups 160, 133, 107, 96, 85, 43 and 37, Seh1, Sec13 and ELYS) in *Homo sapiens* and most metazoans, and it forms a ∼45 nm extended Y-shaped structure^15^. Individual Y-shaped complexes interact in a head-to-tail fashion to form ring-like octameric entities^13^,^16^,^17^, that are integral part of the scaffold^14^. This complex plays a pivotal role in NPC assembly and its absence results in pore-free nuclei with a continuous NE, as shown in studies of depleted *Xenopus laevis* egg extracts^18^,^19^.

Furthermore, Nups have characteristic protein domains^1^,^2^. The α-solenoid/β-propeller containing Nups, for example, are mostly scaffold elements, the transmembrane Nups anchor the NPCs to the NEs, while phenylalanine–glycine (FG) repeat containing Nups interact with cargo–receptor complexes^7^ and constitute the permeability barrier of the central channel^20^.

Acquiring high-resolution structural data on the conformation and gating properties of FG Nups within the confined and crowded central channel is crucial for a deeper understanding of nuclear transport. Since FG repeats are natively unfolded^21^, it is challenging to reveal their detailed architecture. By employing an improved structural analysis of the *X. laevis* NPC in different states of transport, we show that these structures assemble to form a molecular gate at the central channel of the NPC.

## Results

### Highly resolved structure of the native *X. laevis* NPC

**Figure 1:**
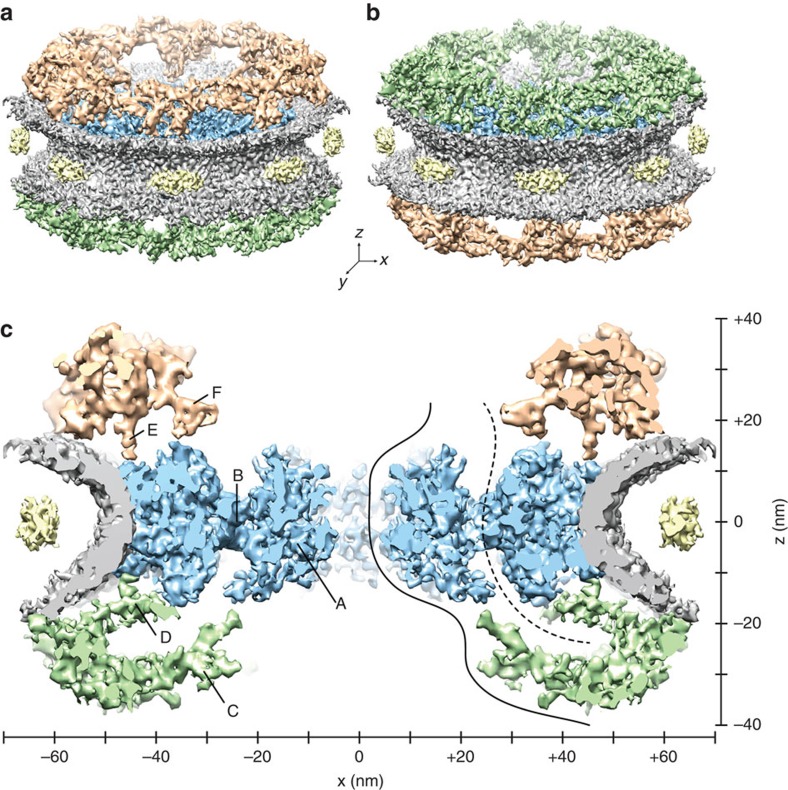
The structure of the native *X. laevis* NPC. (**a**,**b**) Surface-rendered grazing views of the NPC. The NE is depicted in grey, the luminal densities in yellow^12^, and the SR in blue. (**a**) Upper side of the CPR is shown in golden colour. (**b**) Top of the NPR is shown in green colour. (**c**) View of the central nucleocytoplasmic section (25-nm thick) through the NPC structure; CCR (A) and SR-CCR interface (B). Extended linker structures protrude from the NPR (C and D), as well as from the CPR (E). The putative position of the Nup214/Nup88 complexes^14^,^27^ is denoted by F. Suggested nuclear transport routes passing through NPC barrier, illustrated as solid and dashed curves. The axes show the dimensions of the NPC in the *x*- and *y*-direction.

To analyse the structure of the frog NPC, we spread NEs from *X. laevis* oocytes (Supplementary Fig. 1a) directly onto electron microscopy grids and imaged them by cryo-electron tomography^12^,^22^. Out of 64 tomograms, we selected 2,287 subtomograms containing NPCs with evenly distributed orientations. Typical side-views are shown in Supplementary Fig. 1b. We assume that the native NPC structure is well-preserved due to minimal and quick sample preparation and vitrification^23^. Subsequently, subtomograms were subjected to alignment and averaging^24^. The final structure provides an unprecedented view of the native NPC architecture, without any chemical fixation, with up to 20 Å resolution in the most stable regions (Fig. 1, Supplementary Movie 1).

The *X. laevis* NPC is ∼71 nm in height. At the interface between the complex and the NE, the NE spans a luminal space with a height of ∼35 nm and harbours eight luminal densities^12^ that are situated at a diameter of ∼126 nm, encompassing the NPC. The pore itself, formed by the NE, has an inner diameter of ∼90 nm and is filled by a stacked assembly of the three canonical ring moieties (CPR, SR and NPR), as previously described^12^. Although the overall architecture and dimensions of the frog NPC resemble the human NPC^14^, there are major structural differences, mainly in the NPR and central channel.

A comparison between the electron densities of the CPR (Fig. 1a) and the NPR (Fig. 1b) indicates that latter moiety shares only a minor similarity with the CPR. The structure of the CPR appears compact and confined but in contrast to the human NPC structure^14^, the NPR emerges as an entangled filamentous network. This suggests that the C2 symmetry, which was reported for human and yeast NPCs^14^,^20^,^25^, breaks at higher resolution. Consequently, applying C2 symmetrization is not justified here.

### The architecture of the central channel

In Fig. 1c, the central nucleocytoplasmic section (cut in *x–z*-direction, 25 nm thick) through the native NPC structure is displayed. Strikingly, a massive density occupies the central channel (Fig. 1c,A). It exhibits an ordered structure and forms a ring-like assembly. This central channel ring (CCR) is attached to the SR by a porous interface (the SR-CCR interface; Fig. 1c,B), located at a distance of ∼23 nm from the channel centre.

In previous lower resolution NPC structures, the central channel was either masked out^12^,^13^ or, if analysed, shows electron-dense agglomerations around the nucleocytoplasmic axis (*z*-axis) that are connected with the scaffold by weak electron densities^10^,^11^. Indeed, during the initial subtomogram averaging stages, we reproduced these findings and can now assign, at higher resolution, parts of these structures to the CCR (Supplementary Fig. 2a,b).

If cargo complexes always occupy the same regions within the NPC, it would be possible to identify these locations at low resolution, because even though cargo complexes vary in size and shape, in the average, they contribute to common densities. Therefore, the three electron-dense agglomerations that we observe, concentrated at the nucleo- and cytoplasmic entrances and in the centre of the CCR, are presumably caused by a combination of cargo complexes and Nups occupying these locations during nuclear transport, as described before^10^,^11^,^26^ (Supplementary Fig. 2a,b). However, to improve the overall resolution of our final map, we excluded any densities located at a diameter <17 nm around the nucleocytoplasmic axis (see Methods).

The NPR protrudes as an entangled hook-like structure (Fig. 1c,C), which extends inwards and continues along the CCR. Another connection emanates from the basis of the NPR and extends towards the SR (Fig. 1c,D). At the cytoplasmic side, an elongated density bridges the CPR with the SR (Fig. 1c,E). Furthermore, the CPR forms a protrusion (Fig. 1c,F) that was shown to originate from the Nup214/Nup88 complexes^14^,^27^ (Supplementary Fig. 3).

Assuming that cargo–receptor complexes interact with the NPC^7^, we suggest two routes for nuclear transport. The first route is the central route, which proceeds along the inner surfaces of the hook-like structure and the CCR, and subsequently pervades the central pore (Fig. 1c, solid curve, Supplementary Fig. 4, orange tubes). Or alternatively, the second route advances through the NPR meshwork (Supplementary Fig. 4, purple tubes), continues towards the SR, crosses the SR-CCR interface and finally passes the Nup214/Nup88 region (Fig. 1c, dashed curve). Both routes are compatible with the Forest model of NPC architecture^28^.

### Docking of the Y-shaped complex into the density map

To learn more about the scaffold of the NPC, we investigated the position and orientation of the Nup107 complex within the CPR. We used the human Y-shaped complex structure^14^ (Supplementary Fig. 5a) as a model and performed a cross-correlation based search, beginning with 100,000 random starting positions within the asymmetric unit of the CPR. The obtained docking solutions were ordered by their cross-correlation coefficients (redundant fitting results were excluded).

**Figure 2:**
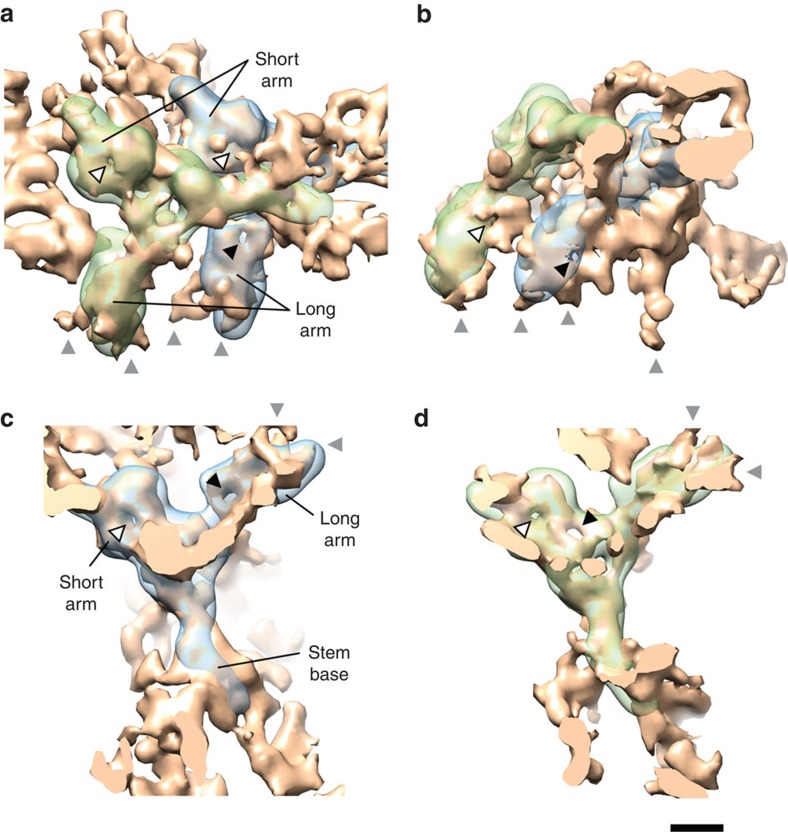
Docking of the Y-shaped complex. The two highest ranked docking solutions of the Y-shaped complex into the CPR electron density (in golden colour) are shown in transparent light blue for the inner position and transparent light green for the outer position. The NE is omitted for clarity, membrane connections are indicated with grey arrowheads. (**a**) Surface-rendered grazing view of the CPR. The putative positions of Seh1 in the inner and outer short arms are marked by white arrowheads. The suggested position of Nup37 in the inner long arm is indicated by a black arrowhead. (**b**) Side view of the CPR asymmetric unit. The proposed position of the Nup160 β-propeller domain in the inner long arm is labelled by a black arrowhead. In addition, a β-propeller-like structure is indicated in the outer long arm by a white arrowhead. (**c**) The CPR is oriented and sectioned such that it shows the electron densities of the inner Y-shaped complex from the top. The assumed positions of Seh1 and Nup37 are marked by white and black arrowheads, respectively. (**d**) The same for the outer Y-shaped complex. The assumed positions of Seh1 and Nup43 are marked by white and black arrowheads, respectively. Scale bar, 5 nm.

Supplementary Fig. 6 summarizes the eight highest ranked docking results. In models 3–8, the long arm of the Y-shaped complex clashes with the Nup214/Nup88 region or the stem base and/or stem tip proceeds outside of the electron density of the CPR (Supplementary Fig. 6, arrows). Because of that, only the two highest ranked solutions are likely. These particular coordinates correspond to the inner and outer positions of the Nup107 complex that were previously described for the human NPC^14^ (Fig. 2a).

Since the radial positions of the N- and/or C-terminal ends of most Nup107 complex members within the NPC are known with high precision from super-resolution microscopy^17^, we used these measurements as an additional restraint for docking. Therefore, we identified the positions of the N-terminal ends of Nup160, Nup107, Nup85, Nup37 and Seh1, as well as the C-terminal end of Nup160 within the Y-shaped complex (Supplementary Fig. 5b), and measured their distances to the radial positions obtained by super-resolution microscopy as a function of docking.

Interestingly, for the highest ranked fit, this analysis yields a mean distance of no more than 4.5 nm±0.6 nm (s.d.) and a maximum distance of ∼5.5 nm for the N terminus of Nup160, in agreement with the linkage error of nanobodies^17^,^29^. However, the lower ranked docking solutions are showing significantly increased deviations compared with the super-resolution microscopy measurements (Supplementary Fig. 6). For example, for the second ranked fit the mean distance is increased to 9.5 nm±1.3 nm and the maximum distance to ∼11.2 nm for the N terminus of Nup85.

Zooming onto the electron densities that were fitted best by the inner and outer position of the Nup107 complex reveals interesting insights into structural changes within the CPR scaffold. First, the two short arms of the Y-shaped complex appear overall similar. Both are showing the characteristic low-resolution feature of a β-propeller (donut-shaped structure) in close proximity to the expected position of Seh1 (Fig. 2a, white arrowheads). However, the two long arms exhibit substantial differences. Nearby to the expected position of Nup37, a β-propeller-like structure emerges in the long arm of the inner Y-shaped complex (Fig. 2a, black arrowhead), whereas this feature is missing in the long arm of the outer Y-shaped complex. Looking from the side towards the two long arm densities emphasizes their differences (Fig. 2b). Here a β-propeller-like structure is observed close to the expected position of the Nup160 β-propeller domain in the inner long arm (Fig. 2b, black arrowhead). Again, this feature is missing in the outer counterpart; however, there, another β-propeller-like structure is shifted towards the top of the CPR (Fig. 2b, white arrowhead).

In Fig. 2c,d the CPR is oriented and sectioned in a way that it allows a direct view of both of the putative Y-shaped complex electron densities. The suggested positions of Seh1 are labelled (Fig. 2c,d, white arrowheads). The proposed position of Nup37 within the inner Y-shaped complex is marked (Fig. 2c, black arrowhead). As seen before from a different perspective, this feature is not present in the outer Y-shaped complex (Fig. 2d). However, in close proximity to the expected position of Nup43, a β-propeller-like structure is observed in the outer Y-shaped complex (Fig. 2d, black arrowhead), which cannot be detected in the inner Y-shaped complex (Fig. 2c).

Given the distinct structural differences between the CPR and the NPR observed here, we were not able to dock the Y-shaped complex into the NPR in any consistent manner.

### Structural changes induced by transcription inhibition

Next, we enhanced the influence of non-transporting states in an averaged structure of the NPC by decreasing the amount of possible transport events. We treated *X. laevis* oocytes with Actinomycin D (ActD), which efficiently inhibits RNA transcription (Supplementary Fig. 7) and therefore RNA export. Moreover, the amount of nuclear proteins, for example, lamin LIII, as well as many others, is reduced (Supplementary Fig. 8). This implies that the mass flux through the NPCs, in both directions, is decreased in ActD-treated *X. laevis* oocytes. From here on, we will refer to NPCs obtained from those samples as ActD-NPCs.

**Figure 3:**
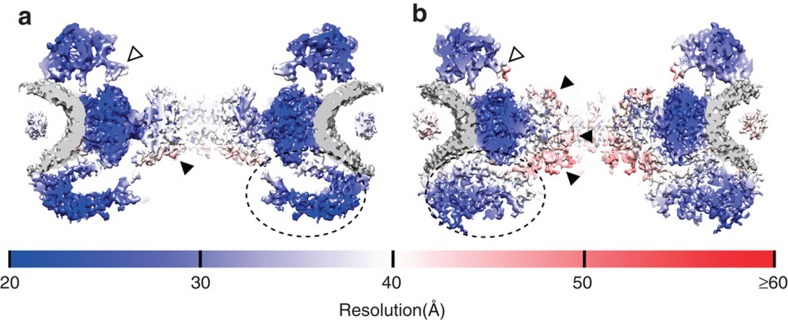
Structural differences in the nucleocytoplasmic section. (**a**) View of the central nucleocytoplasmic section through the native NPC. (**b**) View of the central nucleocytoplasmic section through the ActD-NPC. Both sections are 25-nm thick. The putative Nup214 region is indicated by white arrowheads. The local resolution of the structures is visualized by surface colouring. Resolution values are given by the colour key. The dashed ellipses indicate major structural changes in the NPR. Regions in the CCR with comparatively high structural flexibility are indicated by black arrowheads.

We then performed cryo-electron tomography on NEs purified from *X. laevis* oocytes that were treated with ActD. Out of 71 tomograms, 1,788 NPC containing subtomograms were extracted and subjected to the same processing steps as for the native NPC to obtain an ActD-NPC structure. The nucleocytoplasmic sections of both maps are shown side by side in Fig. 3.

To verify that ActD-NPCs are transport competent, we microinjected lamin B1 encoded mRNA into the cytoplasm and confirmed that efficient nuclear import was conducted (Supplementary Fig. 9a). However, blocking RNA transcription may influence the composition of the permeability barrier of ActD-NPCs. Therefore, we performed a western blot analysis of FG repeat containing Nups (Supplementary Fig. 9b). This experiment shows that all examined FG repeat containing Nups are present in ActD-NPCs. Although similar amounts of Nup62, a FG Nup located in the central channel^1^,^2^, were detected, the levels of the others may have slightly changed. As a consequence, at the putative position of Nup358 (ref. 14) (Supplementary Fig. 10) as well as in the Nup214 region (Fig. 3a,b, white arrowheads) structural changes emerge in the ActD-NPC as compared with the native NPC. However, given the fact that the ActD-NPCs are transport competent and the canonical scaffold moieties are captured as well as the CCR in both NPC structures (Fig. 3), we assume that the largest proportion of changes in the ActD-NPC is related to its reduced transport activity.

In a subtomogram average, nonvariable features are emphasized, whereas variable features smear out or even vanish from the map^10^. Since the degree of structural variability in a certain region is coupled to the attainable resolution, a three-dimensional resolution map allows the interpretation of local resolution depression as increased structural variability^30^. We employed a spatially discretized resolution measurement for both structures (Supplementary Fig. 11), represented by surface colouring in Fig. 3. In both maps, the CPR, SR and NPR were resolved in the range of 20–30 Å. However, the CCR was only resolved to ∼40 Å. This shows that the moieties in the central channel allow for higher structural variability in comparison to the canonical ring moieties of the scaffold. Utilizing that both NPC averages were processed independently^31^, we verified our resolution measurement by cross-resolution between the two maps (Supplementary Figs 11 and 12).

### Structural differences in the NPR and central channel

The most obvious structural differences between the two maps are located in the NPR (Supplementary Movie 2). The extended linker structures at the nucleoplasmic side of the native NPC (Fig. 3a, dashed ellipse) transform into an extended filamentous network in the ActD-NPC (Fig. 3b, dashed ellipse). Therefore, we suggest that the reduced mass flux through the ActD-NPC amplifies the formation of filamentous structures at the NPR.

In the native NPC, the region with the highest structural variability is at the nucleoplasmic entrance to the CCR (Fig. 3a, black arrowhead). In the ActD-NPC, an increase of filamentous entities, with comparatively high structural variability, can be detected within the CCR (Fig. 3b, black arrowheads). Therefore, we propose that the reduced mass flux through the ActD-NPC amplifies the formation of filamentous structures within the CCR with relatively flexible conformation.

**Figure 4:**
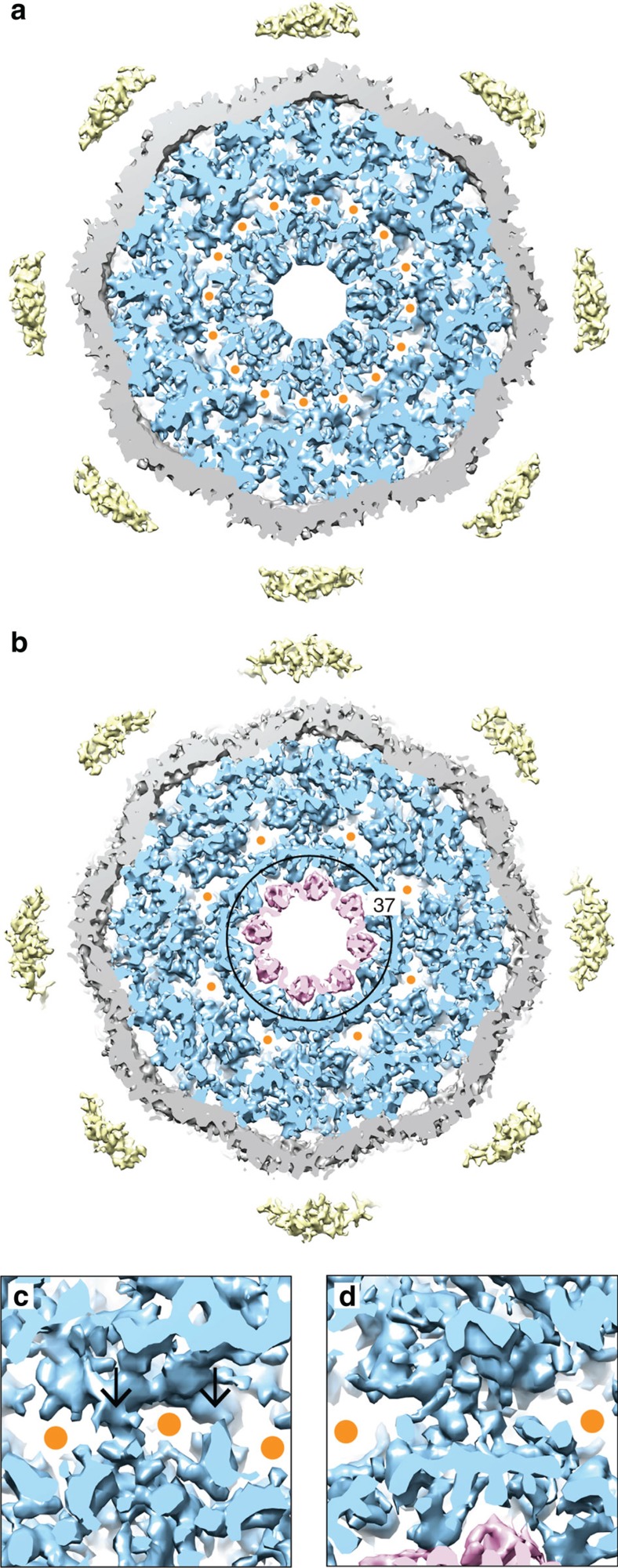
Structural differences in the central channel. (**a**) The central *x–y*-section (10-nm thick) through the native NPC shows the organization of the central channel; the SR and CCR are depicted in blue, the NE in grey and the luminal densities in yellow. Channels marked by orange dots (2 nm in diameter) pass through the SR-CCR interfaces at a radius of ∼23 nm with respect to the centre of the channel (suggested transport route, Fig. 1c, dashed line). (**b**) Due to reduced transport activity, the corresponding view of the ActD-NPC exhibits an intact outer pore ring with a diameter of ∼37 nm. In addition, the inner pore ring adopts a distinct structure (purple). (**c**,**d**) Magnified views (24 × 24 nm^2^) of the SR-CCR interfaces indicate their structural changes. The channel, enclosed by the connecting filaments (arrows) in the native NPC (**c**), is blocked in the ActD-NPC (**d**).

In Fig. 4a,b, the central *x–y*-sections (10-nm thick) through the native NPC and the ActD-NPC are displayed, respectively. Channels of ∼2–6 nm in diameter permeate the sections, in agreement with previous measurements^5^. Alongside the SR-CCR interface, the native NPC structure exhibits 16 equally spaced channels (Fig. 4a, orange dots). Each SR-CCR interface consists of two filaments (Fig. 4c, arrows) connecting the CCR to the SR. In the ActD-NPC, these filaments interact (Fig. 4d), leaving only eight channels open (Fig. 4b, orange dots).

Furthermore, the reduced transport activity through the ActD-NPC induces a structural reorganization of the CCR (Supplementary Movie 3). In particular, its structure exhibits an outer and inner pore ring, where the outer ring possesses an inner diameter of ∼37 nm (Fig. 4b). This value is in good agreement with the maximum diameter of transport cargo^8^. Therefore, we propose that the outer pore ring defines the actual central pore. This would imply that the inner pore ring (Fig. 4b, purple structure) is a part of a structured barrier that seals the central pore.

## Discussion

An improved structure of the NPC is essential for enhancing our understanding of the architecture and function of this giant macromolecular complex. In this work, we present the canonical scaffold moieties of the NPC at molecular resolution^32^, and propose a model for the architecture of the gate at the central channel.

Docking of the Nup107 complex into the CPR confirmed the results obtained for the human NPC^14^. Two Y-shaped complexes reside in one asymmetric unit of the CPR in an inner and outer position. We were able to investigate the two Y-shaped complex structures in greater detail and showed that their conformation^33^ and composition varies substantially *in vivo* (Fig. 2). These differences may explain why only the inner Nup107 complex can be assigned to super-resolution microscopy measurements^17^ with minimal discrepancy (4.5±0.6 nm).

One obvious difference between the human NPC and the frog NPC emerges in the structure of the NPR. Here this part appears as an entangled filamentous network and shows only minor similarity to the CPR (Fig. 1a,b). Therefore, we cannot confirm that there are two Y-shaped complexes in one asymmetric unit of the NPR^14^. From an evolutionary perspective, this difference appears unlikely. Rather, we suggest that the pseudo C2 symmetry of the NPC breaks at higher resolution. As shown in Supplementary Fig. 13, with increasing resolution the differences between the two Y-shaped complex structures in the CPR become more and more apparent and the NPR disintegrates into a filamentous structure. Considering that the structure of the NPR is very sensitive to a reduction of transport events, we propose that this part particularly is a component of the permeability barrier rather than a pure scaffold unit.

Another prominent difference of our *X. laevis* NPC compared with previous studies is the structure inside the central channel (Fig. 1c). Under the assumption that FG Nups fill the central channel^1^,^2^,^20^, the fact that we see the CCR proves immediately that these FG Nups exhibit a certain degree of structural order, otherwise they would be averaged out and not present in the map. At a first glance, this might seem counterintuitive, because FG Nups were suggested to form intrinsically disordered structures^21^. However, in a crowded environment Nups in the central channel may interact with each other^34^ and form assemblies (for example, hydrogels), which can be imaged by electron microscopy^35^,^36^. Therefore, we suggest that the CCR is formed by a cohesive meshwork of FG domains^34^, which allow the passage of cargo–receptor complexes, as shown in the framework of the selective phase model^37^. According to the height of the CCR (∼26 nm) and the diameter of the central pore (∼37 nm), the selective phase could occupy a cylindrical volume of ∼28,000 nm^3^.

On the other hand, the reason we see the CCR is also based on an improvement of the applied subtomogram averaging strategies. For example, in the previous *X. laevis* NPC structure^12^ and in the first human NPC structure^13^ the central channel was simply masked out to reach a decent resolution of the scaffold moieties. With improved averaging methods, a comparable density was found in the central channel of the latest human NPC structure^14^. However, it appears only at lower thresholds, occupies merely ∼25% of the volume of the CCR and is not connected with the SR.

Here we were able to reduce the central channel mask to a diameter of <17 nm. Indeed, the question of how far the CCR protrudes into this still uncharted area cannot be answered based on our maps. However, our map shows that progress in the structural analysis of the NPC is not only a matter of resolution, it is also a matter of completeness (Supplementary Fig. 13, third row). At lower resolution, without employing protomer or even subprotomer averaging techniques (see Methods), it is relatively straightforward to avoid masking of the central channel. Structures inside the central channel were seen, for example, in an early *X. laevis* NPC structure^9^ and in the *Dictyostelium discoideum* NPC structures^10^,^11^. We reproduced these findings and can now assign, at higher resolution and minimal masking, large parts of these structures to the CCR (Supplementary Fig. 2). Furthermore, it seems very likely that the CCR concentrates larger cargo complexes at three regions around the nucleocytoplasmic axis, namely at the nucleoplasmic and cytoplasmic entrances of the CCR and in its centre. This tripartite structure of the central channel was also found in the *D. discoideum* NPC structures^10^,^11^.

Studying the structural response of the NPC to a reduction of transport events showed that both the NPR and CCR reacted by protruding filamentous structures. However, they differ with respect to their structural variability. Both states of the NPR are relatively rigid and were resolved in large parts to molecular resolution (Fig. 3a,b, dashed ellipses), whereas flexibility of the CCR seems to be increased (Fig. 3b, black arrowheads).

To gain additional insight on the gate architecture, we studied NPCs after ActD treatment. Considering that the native NPC and the ActD-NPC share the same overall structure and both are transport competent, it is plausible that the substantial structural changes at the NPR and CCR are due to different transport activity, although minor changes were detected in the FG Nups quantities. Furthermore, since the densities of transport complexes are diluted during higher resolution averaging due to their heterogeneity the structure elements in the final maps can be reliably assigned to NPC components.

We suggest that the changes detected at the NPR resemble the physical properties of FG Nups such as Nup153, which adopts a compact conformation when bound to a transport receptor and a polymer brush-like conformation in its unbound state^38^. Thus, we suggest that the NPR functions as a repulsive entropic barrier at the nucleoplasmic NPC entrance^34^.

**Figure 5:**
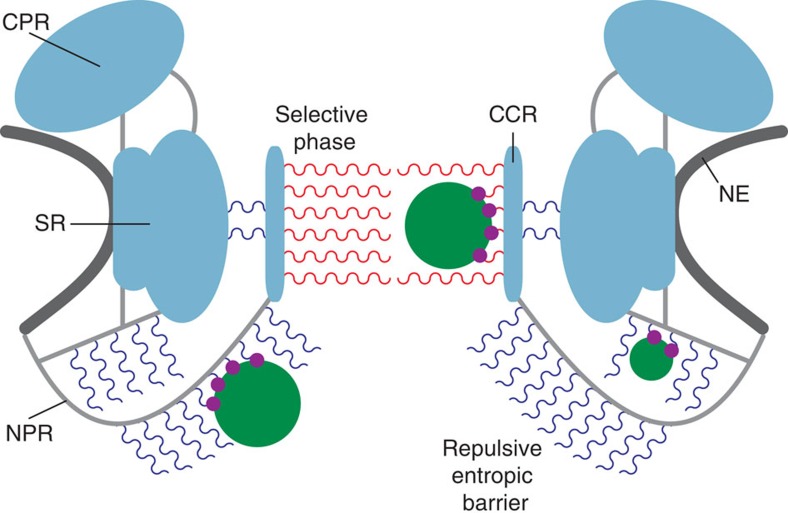
Schematic model of the NPC architecture and gating. The scaffold of the NPC is depicted in cyan and the NE in dark grey. The NPR and linker structures between the ring moieties are illustrated in light grey. FG domains at the NPR construct a repulsive entropic barrier (blue wavy lines), which collapses on interaction with cargo–receptor complexes (depicted as green and purple circles, respectively). The selective phase within the central pore (red wavy lines) exhibits an ordered architecture that is structurally altered during transport.

In summary, we resolved the structure of the NPC in two distinct states with unprecedented resolution and completeness. While some details of the overall architecture and gating mechanism cannot be resolved with certainty and thus remain speculative, a model of NPC architecture and gating that is compatible with our observations is presented in Fig. 5. In the future, a further improvement of subtomogram averaging techniques in combination with state-of-the-art imaging technologies (for example, direct electron detection) will help to further refine our understanding of the structure of the NPC.

## Methods

### Sample preparation

*X. laevis* stage VI oocytes were sorted and the follicular layer was removed manually. Subsequently, the oocytes were stored at 18 °C for no longer than 2 days in modified Barth's saline (10 mM Hepes, pH 7.5, 88 mM NaCl, 1 mM KCl, 0.82 mM MgSO_4_, 0.33 mM Ca(NO_3_)_2_ and 0.41 mM CaCl_2_). Nuclei were manually isolated^39^ and washed in low salt buffer (1 mM KCl and 10 mM Hepes, pH 7.5), prior to spreading onto freshly glow-discharged holey carbon EM grids (R2/1, 200 mesh; Quantifoil, Jena, Germany). Next, 15 nm colloidal gold clusters were applied to the native NE samples before vitrification by rapid plunge freezing in liquid ethane^23^. Furthermore, ActD-treated samples were prepared as described above, although oocytes were incubated for 18 h at 18 °C in modified Barth's saline buffer containing 100 μg ml^−1^ of ActD (Sigma Aldrich, A1410). In addition, low salt buffer containing 7.5 μg ml^−1^ of ActD was used.

The effect of ActD on transcription was verified by western blot analysis. Oocytes were treated with 100 μg ml^−1^ of ActD for 3 h. Next, pEGFP-C1 plasmid containing the human lamin B1 coding sequence was injected into the oocyte nucleus^40^ (15 nl of 10 mg ml^−1^ of Blue Dextran (Sigma Aldrich, D4772-1vl) and 50 ng μl^−1^ plasmid). As a control, the plasmid was injected without any ActD incubation. Thereafter, the oocytes were incubated at 18 °C for 15 h.

For mRNA microinjection, human lamin B1 mRNA was *in vitro* transcribed (AmpliCap-Max T7 High Yield Message Maker Kit; Cellscript C-ACM04037) and purified (RNeasy MiniKit for purification; Qiagen 74104) according to the manufacturer's protocol. The mRNA microinjection was performed after 12 h of ActD treatment (100 μg ml^−1^) and incubated for additional 6 h at 18 °C. Three to six nuclei per condition were extracted and dissolved in pre-heated (95 °C) Laemmli sample buffer (10% glycerol, 3% SDS, 62.5 mM Tris-HCl, 50 mM DTT and 0.05% bromphenol blue).

Next, samples (—three to six nuclei per lane) were loaded onto 10% or 4–12% gradient gels for SDS–PAGE, followed by western blot analysis on polyvinylidene fluoride membranes using anti-lamin B1 antibody (clone M-20, Santa Cruz, #sc-6,217), mab414 (Abcam; ab24609), anti-Lamin LII antibody (clone X223, Santa Cruz, #sc-56,147) and anti-β-actin antibody (Sigma, A5441) as a control.

### Cryo-electron tomography

The data was acquired using Tecnai Polara (FEI, Hillsboro, USA) and FEI Titan Krios transmission electron microscopes equipped with GIF Quantum energy filters and 4 × 4 k UltraScan CCD cameras (Gatan, Pleasanton, USA). Both microscopes were operated at 300 keV in zero-loss mode. Tilt series were recorded using the FEI Xplore3D software. The nominal underfocus was set to −6 μm. The projection images covered an angular range between −60° and 60° with a 3° increment. The cumulative electron dose was kept at 20–60 e^−^ Å^-2^. Tilt series of native NE samples were recorded with a pixel size of 3.3 Å at the specimen level, whereas tilt series of ActD-treated NE samples were recorded with a pixel size of 2.5 Å at the specimen level.

### Contrast transfer function (CTF) correction

The mean defocus was determined by strip-based periodogram averaging for each tilt series^41^. Based on the mean defocus, tilt angle and tilt axis orientation, the defocus gradient for each projection image was calculated. Finally, each projection image was CTF-corrected by phase-flipping according to its defocus gradient^42^. Procedures for CTF correction were implemented in MATLAB (Mathworks, Natick, USA).

### Particle picking and reconstruction

Out of 64 native NE tomograms, 2,287 NPC containing subtomograms were selected manually. Similarly, out of 71 ActD-treated NE tomograms, 1,788 NPC containing subtomograms were selected. The individual NPCs were reconstructed into volumes of 512 × 512 × 512 voxels (native NPCs) or 640 × 640 × 640 voxels (ActD-NPCs). Tilt series alignment and tomographic reconstructions by means of weighted backprojection were carried out with the TOM toolbox software package^43^. Interactive procedures for particle picking were implemented in MATLAB.

### Subtomogram alignment and averaging

As a first step, native NPC and ActD-NPC particles were aligned to a geometrical template, which was constructed from eight spheres (diameter 40 nm) placed on a circle (diameter 90 nm). These initial global alignments, parameterized by three Euler angles (*ϕ*, *θ*, *ψ*) and three translations in *x*-, *y*- and *z-*direction, were verified by approximations of the NPC orientation. These were obtained by fitting the NEs to oblate spheroids and using their normal vector orientations at the respective NPC positions as an estimate for *ϕ* and *θ*. In case of a mismatch (>20°), particles were excluded from further processing.

Subsequently, global alignments were utilized to locate and extract the asymmetric NPC protomers^11^. Here native NPC and ActD-NPC protomer particles were prepared in the same manner. Next, the alignment of the protomers was refined. Therefore, the angular search range was confined to *ϕ*±30°, *θ*±12°, *ψ*±30°. Protomer particles that were assigned to angles at the limits of the search range were excluded from further processing.

In the next step, superfluous top-view protomers were excluded to fine-tune the angular sampling of the averages, and the ActD-NPC protomer average was registered to the native NPC protomer average. Henceforth, modulation transfer function correction was integrated in the alignment procedures^24^. In a refinement step at the protomer level the angular search range was limited to *ϕ*±4°, *θ*±4°, *ψ*±4°.

Subsequently, the asymmetric NPC unit was subdivided into three subprotomers^14^. These subprotomers had a volume of 80 × 80 × 80 voxels at a binning factor of 1 and contained the CPR, the NPR, the SR together with the CCR and overlapping regions. For refinement of the subprotomer alignment, the angular search range was reduced to *ϕ*±2°, *θ*±2°, *ψ*±2°. Furthermore, masks were applied to eliminate the influence of the NE and its lumen. We found that increasing subprotomer volumes negatively affected the attainable resolution. Thus, we excluded densities located at diameters <17 nm during the final refinement step.

The final subprotomer averages were calculated from 5,478 native NPC subprotomers and 3,409 ActD-NPC subprotomers. These particles passed all particle selection steps. Please note that each subtomogram alignment step was finished by adding the new transformations to the previous transformations^24^. Thus, the calculation of an average from the raw NPC particles involves only one trilinear interpolation. Subprotomer averages were arranged to eightfold symmetric models^11^ of the CPR, the NPR and the SR plus the CCR, including a manual segmentation along the overlapping regions. These models were assembled into the final density maps (voxel size of 6.6 Å), namely the structure of the native NPC and the structure of the ActD-NPC. According to the highest local resolution values, the final maps were filtered to a resolution of 20 Å.

Iterative missing wedge-weighted subtomogram alignment and averaging^44^ was performed with the TOM toolbox (tom_corr3d). NE fitting, protomer extraction, protomer subdivision and model building were implemented with MATLAB. Protomer registration and structure visualization were carried out with UCSF Chimera^45^. Segmentations were realized with AMIRA (FEI Visualization Sciences Group, Bordeaux, France).

### Docking

The human Nup107 complex structure (EMD-2443)^14^ was fitted into the CPR by using the global search option of the UCSF Chimera command Fit in map, starting from 100,000 random initial placements within the asymmetric unit of the CPR. In addition, the following crystal structures were fitted into the human Y-shaped complex structure with the UCSF Chimera command Fit in map: PDB-4FHN^46^, PDB-3JRO^47^, PDB-3EWE^48^ and PDB-4I79. Subsequently, the N-terminal ends of Nup160, Nup107, Nup85, Nup37 and Seh1, as well as the C-terminal end of Nup160 were located, marked and transformed according to the found docking solutions with UCSF Chimera. Finally, the distance between the transformed N-terminal/C-terminal positions and their radial positions, obtained by super-resolution microscopy measurements^17^, were evaluated.

### Local resolution measurement

Resolution measurement was spatially discretized. Therefore, the final subprotomer averages were dissected along a regularly spaced grid in subparts, with a grid spacing of 4 × 4 × 4 voxels and a subpart volume of 20 × 20 × 20 voxels. Then, resolution was measured in the subpart volumes, entered into the grid and values in between were extrapolated. In this way, local resolution maps were obtained for the subprotomers, which were subsequently assembled into local resolution maps for the whole NPC. Prior to the local resolution measurement, a spherical mask with softened borders was applied to the subpart volumes. In particular, this mask was controlled to exclude an artificial contribution to the measured resolution^24^.

Here local resolution was measured with two different techniques. First, which was based on conventional Fourier shell correlation^30^, and second that was based on cross-resolution between the two structures, thereby taking advantage of the fact that the native NPC and ActD-NPC subprotomer averages were aligned and averaged independently^31^. In the first case, the 0.5 threshold criterion^49^ was applied, and in the second case the 0.14 threshold criterion^31^. Procedures for local resolution measurement were implemented with MATLAB based on the TOM toolbox.

## Additional information

**Accession codes:** The final subtomogram averages are deposited in the Electron Microscopy Data Bank under the following entries: EMD-3005, EMD-3006, EMD-3007, EMD-3008, EMD-3009, EMD-3010, EMD-3011, and EMD-3012.

**How to cite this article:** Eibauer, M. *et al.* Structure and gating of the nuclear pore complex. *Nat. Commun.* 6:7532 doi: 10.1038/ncomms8532 (2015).

## Supplementary Material

Supplementary Information Supplementary Figures 1-13 and Supplementary References

Supplementary Movie 1 The structure of the native X. laevis NPC

Supplementary Movie 2 Structural changes of the NPR

Supplementary Movie 3 Structural changes of the central channel

## References

[b1] Brohawn S. G., Partridge J. R., Whittle J. R. R. & Schwartz T. U. The nuclear pore complex has entered the atomic age. Structure 17, 1156–1168 (2009). 19748337 10.1016/j.str.2009.07.014 PMC2776643

[b2] Hoelz A., Debler E. W. & Blobel G. The structure of the nuclear pore complex. Annu. Rev. Biochem. 80, 613–643 (2011). 21495847 10.1146/annurev-biochem-060109-151030

[b3] Reichelt R. *et al.* Correlation between structure and mass distribution of the nuclear pore complex and of distinct pore complex components. J. Cell Biol. 110, 883–894 (1990). 2324201 10.1083/jcb.110.4.883 PMC2116066

[b4] Cronshaw J. M., Krutchinsky A. N., Zhang W., Chait B. T. & Matunis M. J. Proteomic analysis of the mammalian nuclear pore complex. J. Cell Biol. 158, 915–927 (2002). 12196509 10.1083/jcb.200206106 PMC2173148

[b5] Mohr D., Frey S., Fischer T., Guettler T. & Goerlich D. Characterization of the passive permeability barrier of nuclear pore complexes. EMBO J. 28, 2541–2553 (2009). 19680228 10.1038/emboj.2009.200 PMC2728435

[b6] Fried H. & Kutay U. Nucleocytoplasmic transport: taking an inventory. Cell. Mol. Life Sci. 60, 1659–1688 (2003). 14504656 10.1007/s00018-003-3070-3 PMC11138860

[b7] Rexach M. & Blobel G. Protein import into nuclei: association and dissociation reactions involving transport substrate, transport factors, and nucleoporins. Cell 83, 683–692 (1995). 8521485 10.1016/0092-8674(95)90181-7

[b8] Pante N. & Kann M. Nuclear pore complex is able to transport macromolecules with diameters of ∼39 nm. Mol. Biol. Cell 13, 425–434 (2002). 11854401 10.1091/mbc.01-06-0308 PMC65638

[b9] Akey C. W. & Radermacher M. Architecture of the *Xenopus* nuclear pore complex revealed by three-dimensional cryo-electron microscopy. J. Cell Biol. 122, 1–19 (1993). 8314837 10.1083/jcb.122.1.1 PMC2119598

[b10] Beck M. *et al.* Nuclear pore complex structure and dynamics revealed by cryoelectron tomography. Science 306, 1387–1390 (2004). 15514115 10.1126/science.1104808

[b11] Beck M., Lucic V., Foerster F., Baumeister W. & Medalia O. Snapshots of nuclear pore complexes in action captured by cryo-electron tomography. Nature 449, 611–615 (2007). 17851530 10.1038/nature06170

[b12] Frenkiel-Krispin D., Maco B., Aebi U. & Medalia O. Structural analysis of a metazoan nuclear pore complex reveals a fused concentric ring architecture. J. Mol. Biol. 395, 578–586 (2010). 19913035 10.1016/j.jmb.2009.11.010

[b13] Maimon T., Elad N., Dahan I. & Medalia O. The human nuclear pore complex as revealed by cryo-electron tomography. Structure 20, 998–1006 (2012). 22632834 10.1016/j.str.2012.03.025

[b14] Bui K. H. *et al.* Integrated structural analysis of the human nuclear pore complex scaffold. Cell 155, 1233–1243 (2013). 24315095 10.1016/j.cell.2013.10.055

[b15] Lutzmann M., Kunze R., Buerer A., Aebi U. & Hurt E. Modular self-assembly of a Y-shaped multiprotein complex from seven nucleoporins. EMBO J. 21, 387–397 (2002). 11823431 10.1093/emboj/21.3.387 PMC125826

[b16] Kampmann M., Atkinson C. E., Mattheyses A. L. & Simon S. M. Mapping the orientation of nuclear pore proteins in living cells with polarized fluorescence microscopy. Nat. Struct. Biol. 18, 643–649 (2011). 10.1038/nsmb.2056 PMC3109191 21499242

[b17] Szymborska A. *et al.* Nuclear pore scaffold structure analysed by super-resolution microscopy and particle averaging. Science 341, 655–658 (2013). 23845946 10.1126/science.1240672

[b18] Walther T. C. *et al.* The conserved Nup107-160 complex is critical for nuclear pore complex assembly. Cell 113, 195–206 (2003). 12705868 10.1016/s0092-8674(03)00235-6

[b19] Harel A. *et al.* Removal of a single pore subcomplex results in vertebrate nuclei devoid of nuclear pores. Mol. Cell 11, 853–864 (2003). 12718872 10.1016/s1097-2765(03)00116-3

[b20] Rout M. P. *et al.* The yeast nuclear pore complex: composition, architecture, and transport mechanism. J. Cell Biol. 148, 635–651 (2000). 10684247 10.1083/jcb.148.4.635 PMC2169373

[b21] Denning D. P., Patel S. S., Uversky V., Fink A. L. & Rexach M. Disorder in the nuclear pore complex: the FG repeat regions of nucleoporins are natively unfolded. Proc. Natl Acad. Sci. USA 100, 2450–2455 (2003). 12604785 10.1073/pnas.0437902100 PMC151361

[b22] Lucic V., Foerster F. & Baumeister W. Structural studies by electron tomography: from cells to molecules. Annu. Rev. Biochem. 74, 833–865 (2005). 15952904 10.1146/annurev.biochem.73.011303.074112

[b23] Dubochet J. *et al.* Cryo-electron microscopy of vitrified specimens. Q. Rev. Biophys. 21, 129–228 (1988). 3043536 10.1017/s0033583500004297

[b24] Eibauer M. *et al.* Unraveling the structure of membrane proteins *in situ* by transfer function corrected cryo-electron tomography. J. Struct. Biol. 180, 488–496 (2012). 23000705 10.1016/j.jsb.2012.09.008

[b25] Alber F. *et al.* The molecular architecture of the nuclear pore complex. Nature 450, 695–701 (2007). 18046406 10.1038/nature06405

[b26] Stoffler D. *et al.* Cryo-electron tomography provides novel insights into nuclear pore architecture: Implications for nucleocytoplasmic transport. J. Mol. Biol. 328, 119–130 (2003). 12684002 10.1016/s0022-2836(03)00266-3

[b27] Gaik M. *et al.* Structural basis for assembly and function of the Nup82 complex in the nuclear pore scaffold. J. Cell Biol. 208, 283–297 (2015). 25646085 10.1083/jcb.201411003 PMC4315244

[b28] Yamada J. *et al.* A bimodal distribution of two distinct categories of intrinsically disordered structures with separate functions in FG nucleoporins. Mol. Cell. Proteomics 9, 2205–2224 (2010). 20368288 10.1074/mcp.M000035-MCP201 PMC2953916

[b29] Ries J., Kaplan C., Platonova E., Eghlidi H. & Ewers H. A simple, versatile method for GFP-based super-resolution microscopy via nanobodies. Nat. Methods 9, 582–584 (2012). 22543348 10.1038/nmeth.1991

[b30] Beck F. *et al.* Near-atomic resolution structural model of the yeast 26S proteasome. Proc. Natl Acad. Sci. USA 109, 14870–14875 (2012). 22927375 10.1073/pnas.1213333109 PMC3443124

[b31] Scheres S. H. W. & Chen S. Prevention of overfitting in cryo-EM structure determination. Nat. Methods 9, 853–854 (2012). 22842542 10.1038/nmeth.2115 PMC4912033

[b32] Robinson C. V., Sali A. & Baumeister W. The molecular sociology of the cell. Nature 450, 973–982 (2007). 18075576 10.1038/nature06523

[b33] Kampmann M. & Blobel G. Three-dimensional structure and flexibility of a membrane-coating module of the nuclear pore complex. Nat. Struct. Biol. 16, 782–788 (2009). 10.1038/nsmb.1618 PMC2706296 19503077

[b34] Patel S. S., Belmont B. J., Sante J. M. & Rexach M. F. Natively unfolded nucleoporins gate protein diffusion across the nuclear pore complex. Cell 129, 83–96 (2007). 17418788 10.1016/j.cell.2007.01.044

[b35] Milles S. *et al.* Facilitated aggregation of FG nucleoporins under molecular crowding conditions. EMBO Rep. 14, 178–183 (2013). 23238392 10.1038/embor.2012.204 PMC3596131

[b36] Estroff L. A., Leiserowitz L., Addadi L., Weiner S. & Hamilton A. D. Characterization of an organic hydrogel: A cryo-transmission electron microscopy and x-ray diffraction study. Adv. Mater. 15, 38–42 (2003).

[b37] Frey S. & Goerlich D. A saturated FG-repeat hydrogel can reproduce the permeability properties of nuclear pore complexes. Cell 130, 512–523 (2007). 17693259 10.1016/j.cell.2007.06.024

[b38] Lim R. Y. H. *et al.* Nanomechanical basis of selective gating by the nuclear pore complex. Science 318, 640–643 (2007). 17916694 10.1126/science.1145980

[b39] Jarnik M. & Aebi U. Toward a more complete 3-D structure of the nuclear pore complex. J. Struct. Biol. 107, 291–308 (1991). 1725493 10.1016/1047-8477(91)90054-z

[b40] Stick R. & Goldberg M. W. Oocytes as an experimental system to analyse the ultrastructure of endogenous and ectopically expressed nuclear envelope components by field-emission scanning electron microscopy. Methods 51, 170–176 (2010). 20085817 10.1016/j.ymeth.2010.01.015

[b41] Fernandez J. J., Li S. & Crowther R. A. CTF determination and correction in electron cryotomography. Ultramicroscopy 106, 587–596 (2006). 16616422 10.1016/j.ultramic.2006.02.004

[b42] Zanetti G., Riches J. D., Fuller S. D. & Briggs J. A. G. Contrast transfer function correction applied to cryo-electron tomography and sub-tomogram averaging. J. Struct. Biol. 168, 305–312 (2009). 19666126 10.1016/j.jsb.2009.08.002 PMC2806944

[b43] Nickell S. *et al.* TOM software toolbox: acquisition and analysis for electron tomography. J. Struct. Biol. 149, 227–234 (2005). 15721576 10.1016/j.jsb.2004.10.006

[b44] Foerster F., Medalia O., Zauberman N., Baumeister W. & Fass D. Retrovirus envelope protein complex structure *in situ* studied by cryo-electron tomography. Proc. Natl Acad. Sci. USA 102, 4729–4734 (2005). 15774580 10.1073/pnas.0409178102 PMC555690

[b45] Pettersen E. F. *et al.* UCSF Chimera—a visualization system for exploratory research and analysis. J. Comput. Chem. 25, 1605–1612 (2004). 15264254 10.1002/jcc.20084

[b46] Bilokapic S. & Schwartz T. U. Molecular basis for Nup37 and ELY5/ELYS recruitment to the nuclear pore complex. Proc. Natl Acad. Sci. USA 109, 15241–15246 (2012). 22955883 10.1073/pnas.1205151109 PMC3458321

[b47] Brohawn S. G. & Schwartz T. U. Molecular architecture of the Nup84-Nup145C-Sec13 edge element in the nuclear pore complex lattice. Nat. Struct. Biol. 16, 1173–1177 (2009). 10.1038/nsmb.1713 PMC3398507 19855394

[b48] Brohawn S. G., Leksa N. C., Spear E. D., Rajashankar K. R. & Schwartz T. U. Structural evidence for common ancestry of the nuclear pore complex and vesicle coats. Science 322, 1369–1373 (2008). 18974315 10.1126/science.1165886 PMC2680690

[b49] Van Heel M. & Schatz M. Fourier shell correlation threshold criteria. J. Struct. Biol. 151, 250–262 (2005). 16125414 10.1016/j.jsb.2005.05.009

