# Supplementary material for: Structure and gating of the nuclear pore complex
Source: Nat Commun. 2015 Jun 26;6:7532. doi: 10.1038/ncomms8532 (PMC4491817; doi:10.1038/ncomms8532)
Supplement: Supplementary Information — Supplementary Figures 1-13 and Supplementary References [file ncomms8532-s1.pdf]

Structure and gating of the nuclear pore complex.

Authors: Matthias Eibauer, Mauro Pellanda, Yagmur Turgay, Anna Dubrovsky, Annik Wild, Ohad Medalia

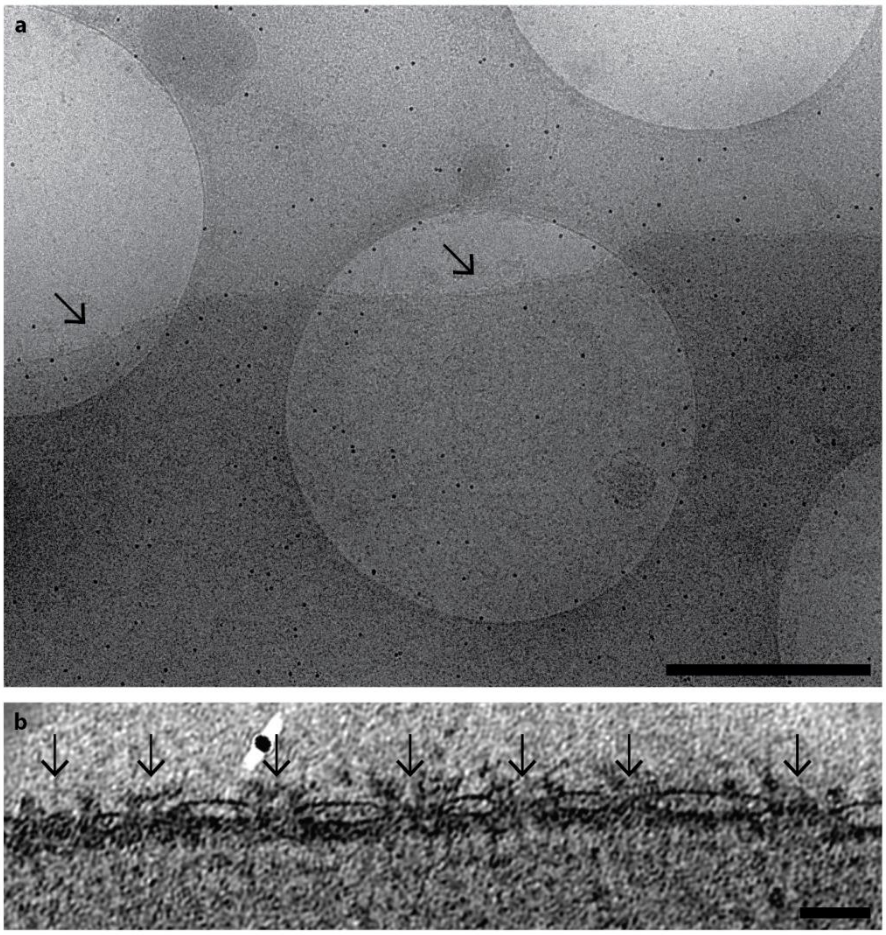

Supplementary Figure 1 | Cryo-electron tomography of spread *X. laevis* oocyte NEs. (**a**) Transmission electron micrograph of a vitrified NE (arrows) spread over an electron microscopy grid. Scale bar indicates 1 µm. (**b**) The image shows a 2.64 nm thick x-y-slice through a typical tomogram. The arrows point to NPCs in a side-view orientation. Scale bar indicates 100 nm.

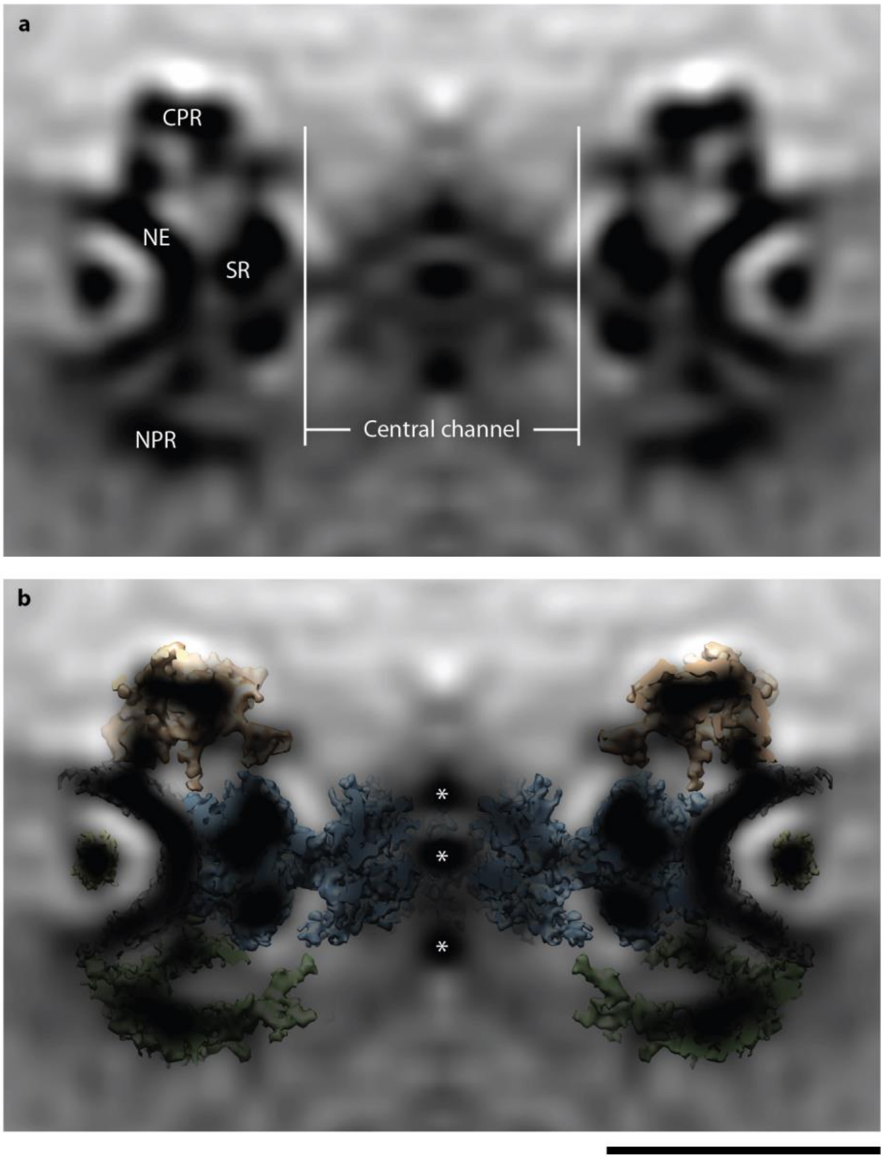

Supplementary Figure 2 | The nucleocytoplasmic plane at low resolution. (**a**) The image shows the nucleocytoplasmic plane (2.64 nm thick x-z-slice) of the frog NPC at low resolution (~7 nm). The weak electron densities in the central channel appear as a part of the CCR in the final structure. (**b**) Overlay of the final structure with the nucleocytoplasmic plane at low resolution. Three electron-dense agglomerations (marked by asterisks) are concentrated at the nucleo- and cytoplasmic entrances and in the center of the CCR. These densities are over-represented due to the rotational averaging but may represent cargo complexes caught in a static position during transport. Scale bar indicates 50 nm.

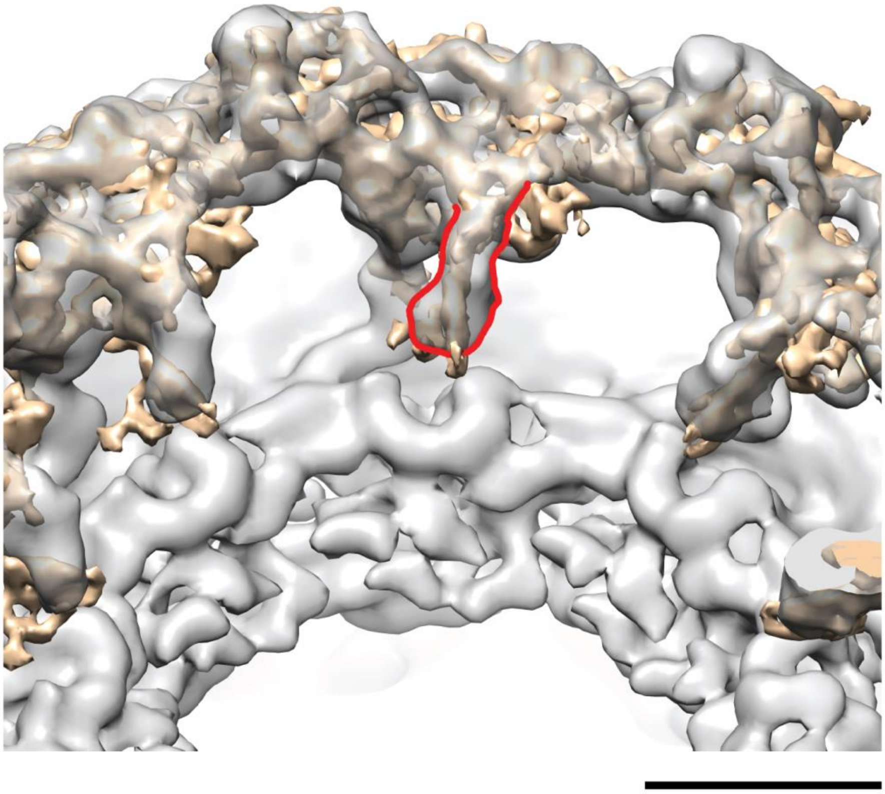

Supplementary Figure 3 | Localization of the Nup214/Nup88 complexes. The image shows the structure of the human NPC (EMD-2444)[1] in transparent grey. The putative position of the human Nup214/Nup88 complexes is outlined in red[1,2]. The frog CPR (golden color) is docked into the human CPR. The position of the density, labelled with F in Fig. 1c, matches with the localization of the human Nup214/Nup88 complexes. Scale bar indicates 20 nm.

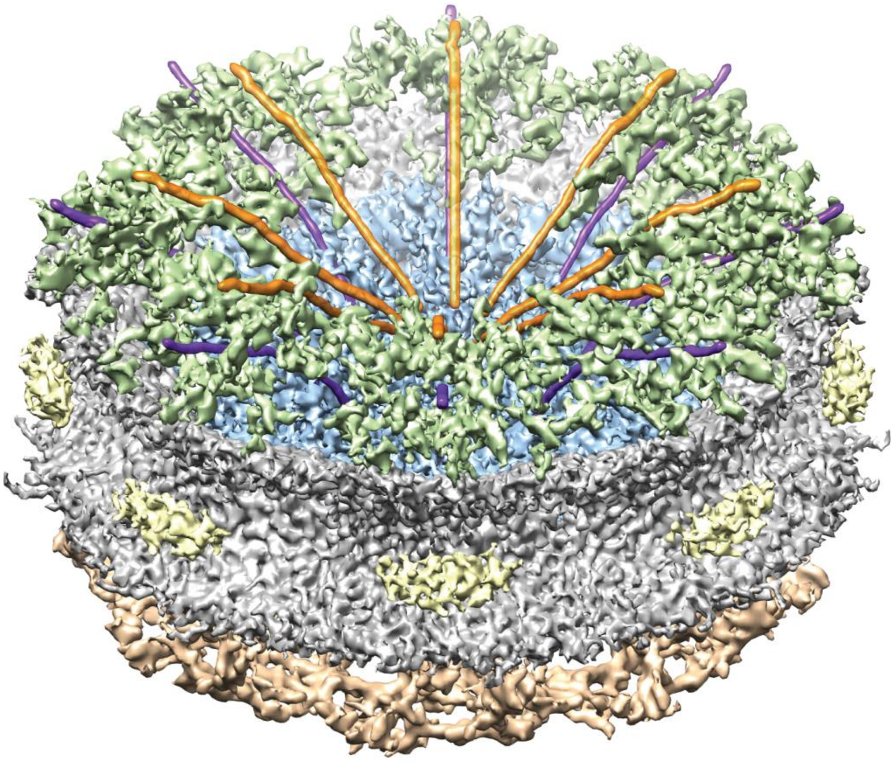

Supplementary Figure 4 | Possible transport routes across the NPC. The central route is indicated by orange tubes. An alternative route is traced by purple tubes. The NE is depicted grey. Luminal densities are colored yellow. The NPR is rendered green, the SR blue, and the CPR golden.

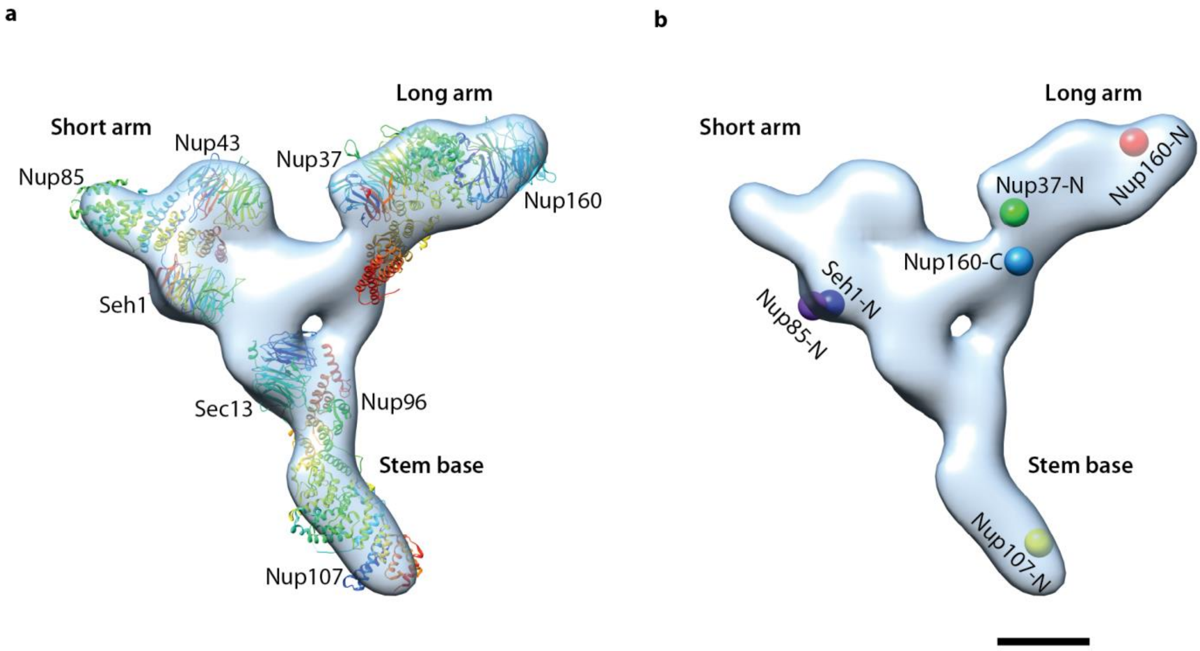

Supplementary Figure 5 | The Y-shaped complex. (**a**) The vertex structure (Short arm, long arm, and stem base) of the human Nup107 complex (EMD-2443)[1] is shown in light blue. We used this structure for docking. The stem tip, which prolongs the stem base, is not contained in the map, because it was averaged out due to high flexibility[1]. The crystal structures of Nup160/Nup37 (PDB-4FHN)[3], Nup96/Sec13/Nup107 (PDB-3JRO)[4], Nup85/Seh1 (PDB-3EWE)[5], and Nup43 (PDB-4I79) were fitted into the vertex structure. (**b**) Based on the fitting the N-terminal ends of Nup160, Nup107, Nup85, Nup37, and Seh1, as well as the C-terminal end of Nup160 were located (marked by colored spheres). Scale bar indicates 5 nm.

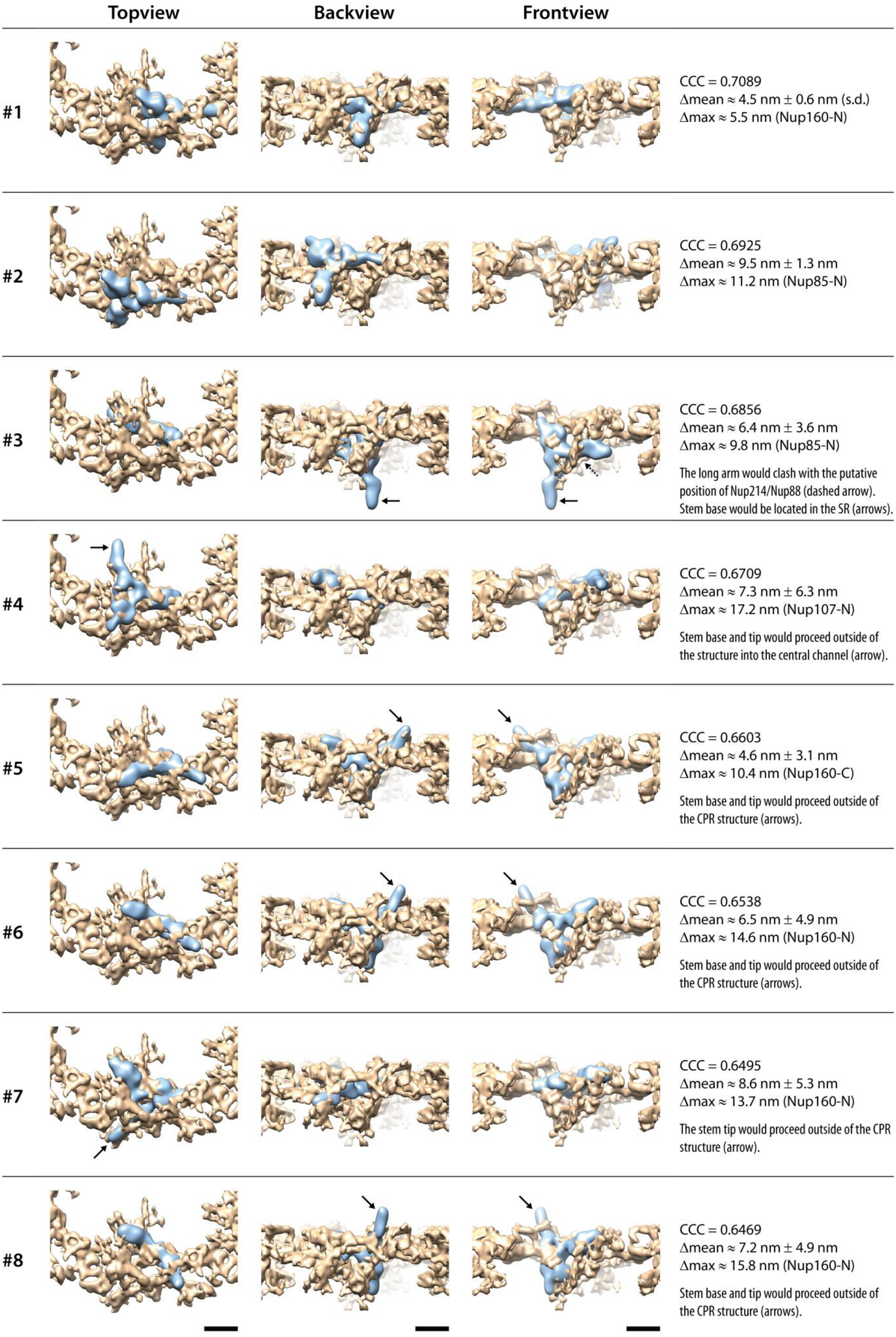
Topview
Backview
Frontview
#1
CCC = 0.7089
Δmean ≈ 4.5 nm ± 0.6 nm (s.d.)
Δmax ≈ 5.5 nm (Nup160-N)
#2
CCC = 0.6925
Δmean ≈ 9.5 nm ± 1.3 nm
Δmax ≈ 11.2 nm (Nup85-N)
#3
CCC = 0.6856
Δmean ≈ 6.4 nm ± 3.6 nm
Δmax ≈ 9.8 nm (Nup85-N)
The long arm would clash with the putative position of Nup214/Nup88 (dashed arrow). Stem base would be located in the SR (arrows).
#4
CCC = 0.6709
Δmean ≈ 7.3 nm ± 6.3 nm
Δmax ≈ 17.2 nm (Nup107-N)
Stem base and tip would proceed outside of the structure into the central channel (arrow).
#5
CCC = 0.6603
Δmean ≈ 4.6 nm ± 3.1 nm
Δmax ≈ 10.4 nm (Nup160-C)
Stem base and tip would proceed outside of the CPR structure (arrows).
#6
CCC = 0.6538
Δmean ≈ 6.5 nm ± 4.9 nm
Δmax ≈ 14.6 nm (Nup160-N)
Stem base and tip would proceed outside of the CPR structure (arrows).
#7
CCC = 0.6495
Δmean ≈ 8.6 nm ± 5.3 nm
Δmax ≈ 13.7 nm (Nup160-N)
The stem tip would proceed outside of the CPR structure (arrow).
#8
CCC = 0.6469
Δmean ≈ 7.2 nm ± 4.9 nm
Δmax ≈ 15.8 nm (Nup160-N)
Stem base and tip would proceed outside of the CPR structure (arrows).

Supplementary Figure 6 | Summary of docking solutions. The eight highest ranked docking solutions are displayed. The CPR is displayed in golden color and the Y-shaped complex in blue. In the first column, the CPR is viewed from the top, in the second from the back, and in the third from the central channel. The docking solutions are ordered according to their cross-correlation coefficients (CCC; given in the fourth column). The position of the N-terminal ends of Nup160, Nup107, Nup85, Nup37, and Seh1, as well as the C-terminal end of Nup160 were measured as a function of docking and the distance to their radial positions obtained by super-resolution microscopy[6] were evaluated. The mean distance and the standard deviation are given in the fourth column (Δmean), as well as the highest distance (Δmax) and the name of the Nup terminal end associated with Δmax. The comments in the fourth column correspond to the black arrows. Scale bars indicate 10 nm.

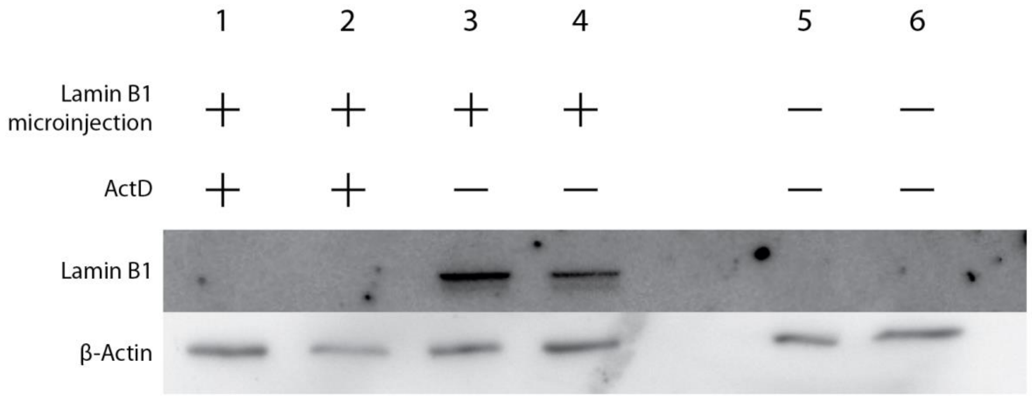

Supplementary Figure 7 | ActD inhibits RNA transcription in *X. laevis* oocytes. Western blot analysis of *X. laevis* nuclei microinjected with a plasmid encoding for human lamin B1 as a reporter (lanes 1-4) and non-microinjected as a control (lanes 5 and 6). The oocytes were incubated with ActD for 18 h at 18 °C (lanes 1 and 2). Lamin B1 protein production was abolished. β-actin served as loading control.

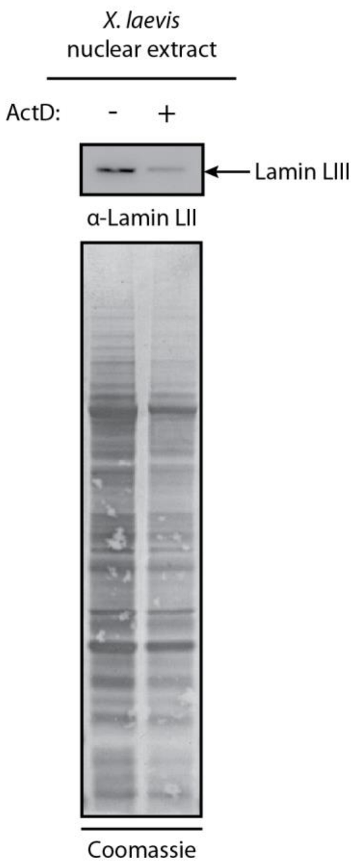

Supplementary Figure 8 | ActD treatment results in a decrease of total protein levels in *X. laevis* nuclei. Western blot analysis of *X. laevis* nuclei from oocytes after treatment with ActD for 18 h at 18 °C. Immunostaining (top) using α-Lamin LII antibodies shows a decrease of endogenous *X. laevis* lamin LIII protein levels and Coomassie stained membrane (bottom) displays the decrease of the total protein levels after ActD treatment.

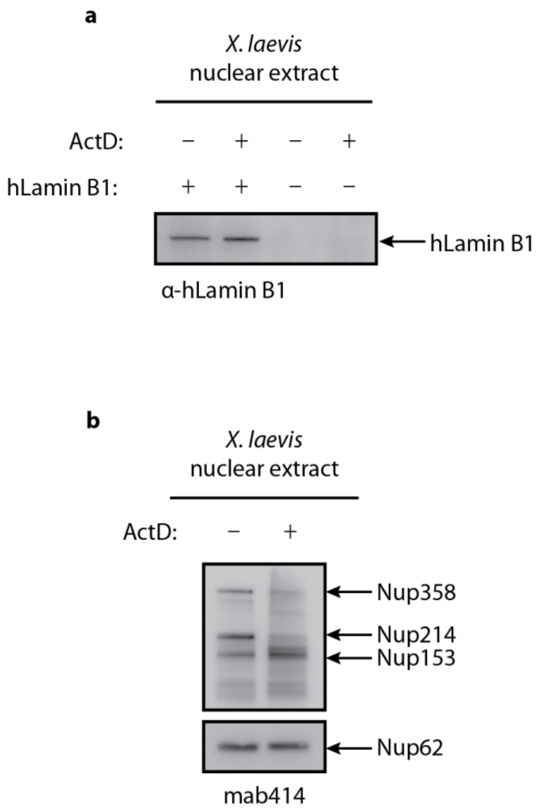

Supplementary Figure 9 | Transport competence and composition of ActD-NPCs. (**a**) Transport competence was validated using α-human lamin B1 antibodies to detect ectopically expressed human Lamin B1 in *X. laevis* nuclei after microinjection of human lamin B1 mRNA into ActD or buffer pre-treated (12 h) oocytes. Nuclei extraction and SDS sample preparation was performed 6 h after microinjection. Western blot analysis displays equal amounts of human lamin B1 after ActD treatment. (**b**) Western blot analysis of *X. laevis* nuclei from oocytes after treatment with ActD for 18 h at 18 °C. Immunostaining using mab414 shows the protein levels of the endogenous *X. laevis* nucleoporins Nup358, Nup214, Nup153, and Nup62.

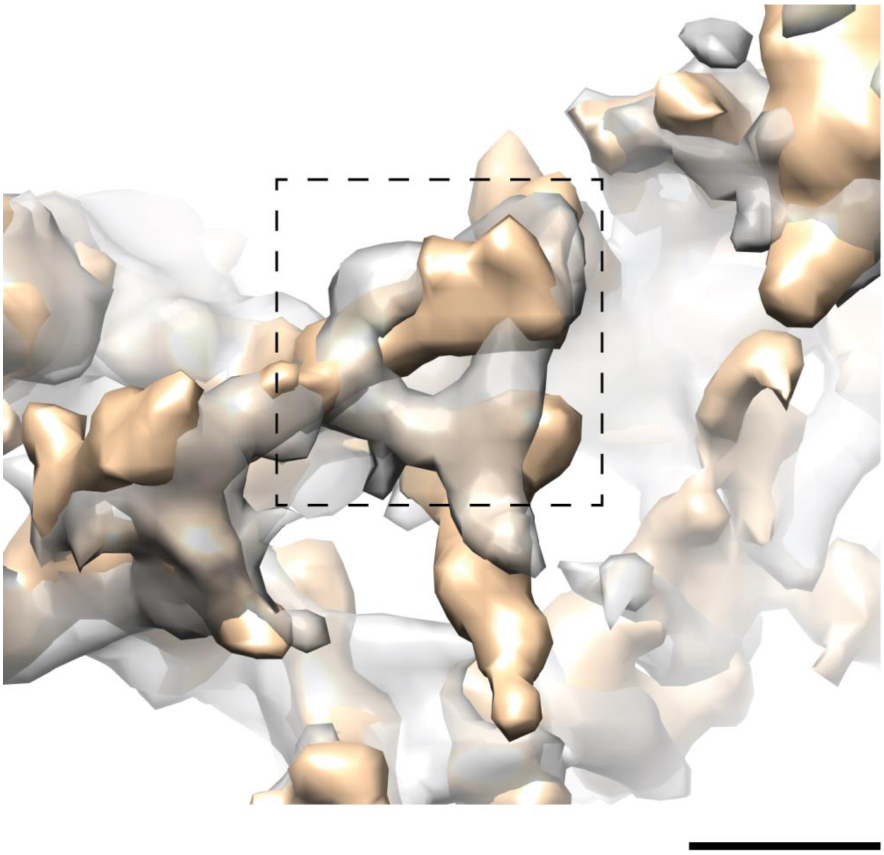

Supplementary Figure 10 | Structural differences in the Nup358 region. The image shows a top view of the CPR. The native structure is rendered transparent grey and the ActD treated structure in golden color. The dashed box indicates the putative Nup358 region[1]. Within this region structural differences appear between the two CPR structures. Scale bar indicates 5 nm.

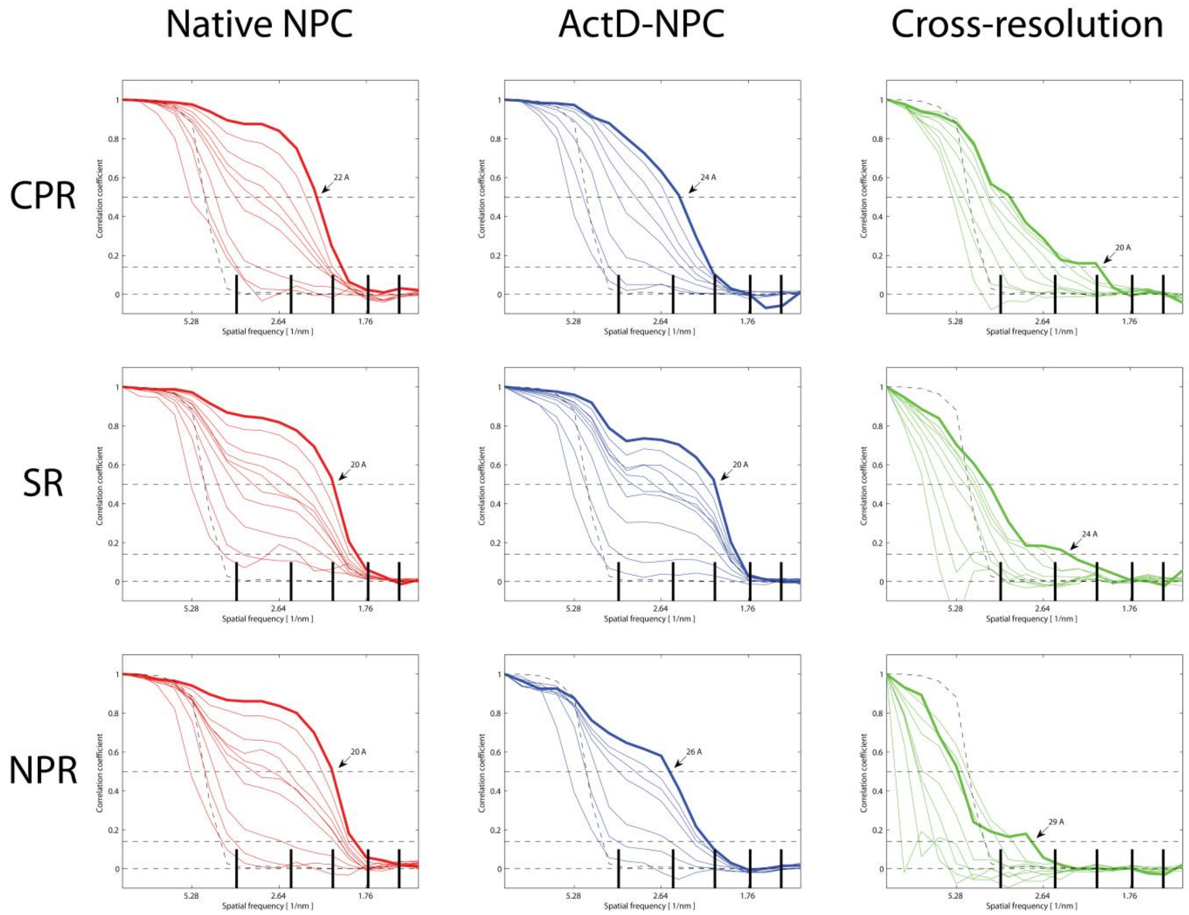

Supplementary Figure 11 | Spatially discretized resolution analysis. Plots in the first row are related to the CPR, second row to the SR, and third row to the NPR. Plots in the first and second column are based on resolution measurement by Fourier shell correlation. Plots in the third column are based on cross-resolution measurement between the native NPC structure and the ActD-NPC structure. In contrast to mapping each local resolution value on the surface of the structures, as shown in Fig. 3 and Supplementary Fig. 12, the local Fourier shell correlation curves indicating the same resolution value were averaged and plotted. Thereby, the 0.5 threshold criterion was used for the conventional resolution measurement[7] and the 0.14 criterion in the cross-resolution case, taking advantage of the fact that the native NPC and the ActD-NPC were independently aligned and averaged[8]. As a masking control,

resolution measurement was applied to noise volumes[9] (dashed curves). Solid vertical line segments indicate zeros of a CTF with a defocus of -6.2 µm.

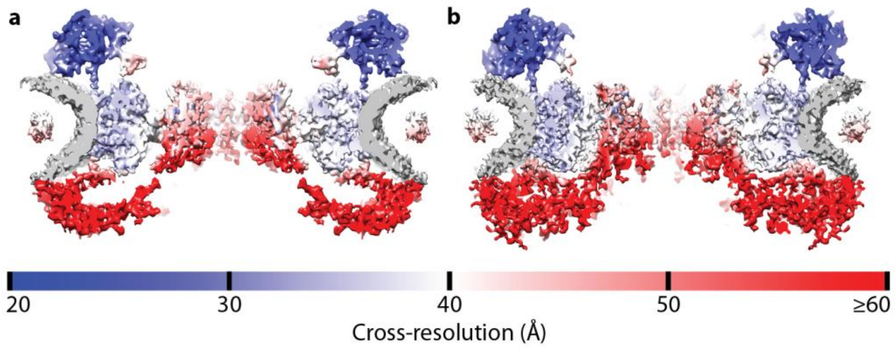

Supplementary Figure 12 | Cross-resolution measurement. (**a**) View of the central nucleocytoplasmic section through the native NPC. (**b**) View of the central nucleocytoplasmic section through the ActD-NPC. Both sections are 25 nm thick. The local cross-resolution values of the structures are visualized by surface coloring. Resolution values are given by the color key. In the CPR and SR the resolution values measured by cross-resolution are similar to the resolution values obtained by conventional resolution measurement. Due to the distinct structural differences between the native NPC and the ActD-NPC in the NPR and the CCR the cross-resolution drops in these regions.

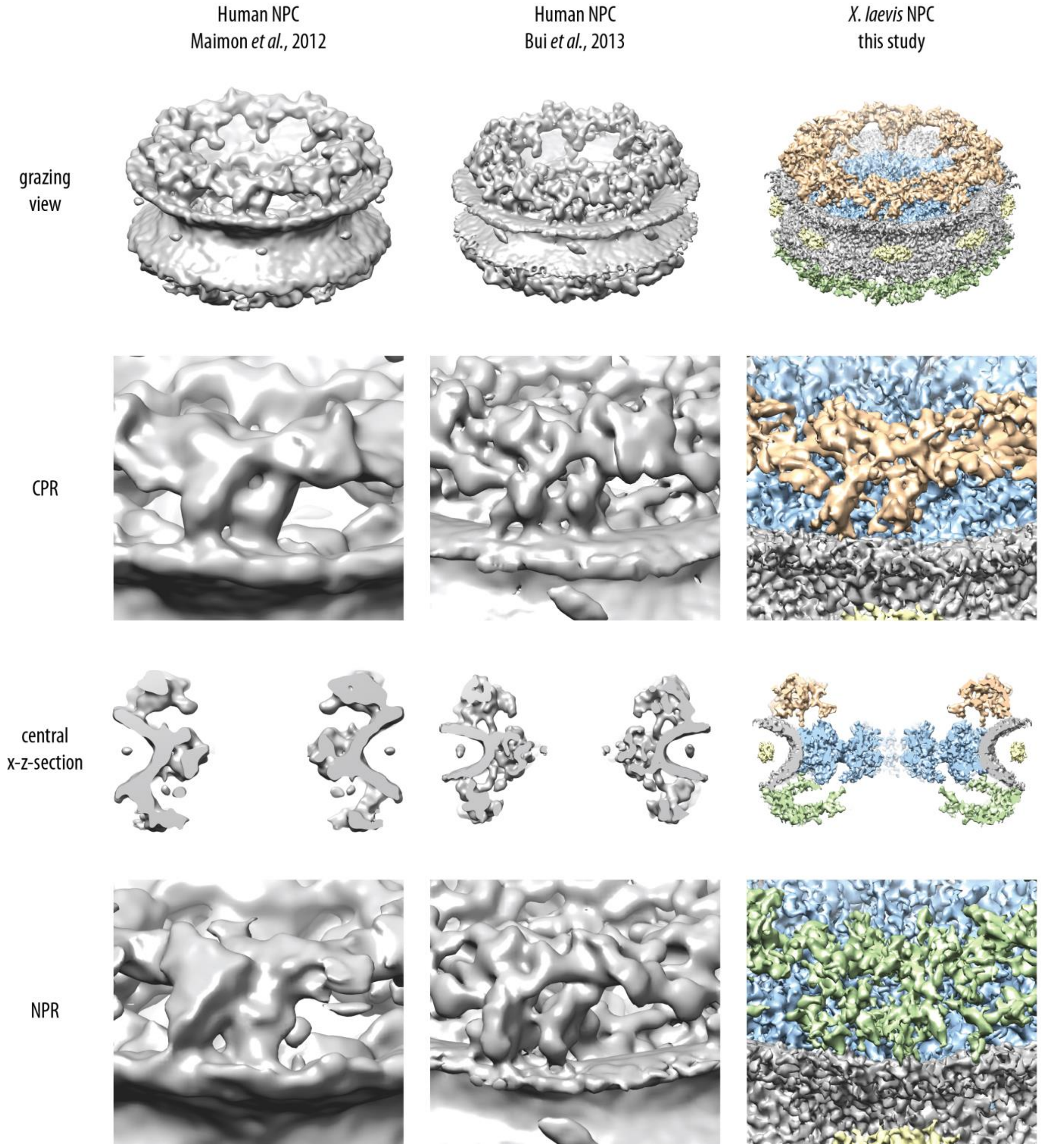

Supplementary Figure 13 | Comparison between higher eukaryote NPCs. The structures are ordered from left to right with increasing resolution and completeness (first column, human NPC[10], EMD-5411, 66 Å resolution; second column, human NPC[1], EMD-2444, 35 Å resolution; third column, *X. laevis* NPC, this study). In each row the images show the respective views of the whole NPC, the CPR, the central x-z-section, and the NPR.

Supplementary References:

1. Bui, K. H. *et al.* Integrated structural analysis of the human nuclear pore complex scaffold. *Cell* **155,** 1233-1243 (2013).
2. Gaik, M. *et al.* Structural basis for assembly and function of the Nup82 complex in the nuclear pore scaffold. *J. Cell Biol.* **208**, 283-297 (2015).
3. Bilokapic, S. & Schwartz, T. U. Molecular basis for Nup37 and ELY5/ELYS recruitment to the nuclear pore complex. *Proc. Natl. Acad. Sci. U.S.A.* **109,** 15241-15246 (2012).
4. Brohawn, S. G. & Schwartz, T. U. Molecular architecture of the Nup84-Nup145C-Sec13 edge element in the nuclear pore complex lattice. *Nature Struct. Biol.* **16,** 1173-1177 (2009).
5. Brohawn, S. G., Leksa, N. C., Spear, E. D., Rajashankar, K. R. & Schwartz, T. U. Structural evidence for common ancestry of the nuclear pore complex and vesicle coats. *Science* **322**, 1369-1373 (2008).
6. Szymborska, A. *et al.* Nuclear pore scaffold structure analyzed by super-resolution microscopy and particle averaging. *Science* **341,** 655-658 (2013).
7. Van Heel, M. & Schatz, M. Fourier shell correlation threshold criteria. *J. Struct. Biol.* **151**, 250-262 (2005).
8. Scheres, S. H. W. & Chen, S. Prevention of overfitting in cryo-EM structure determination. *Nature Methods* **9,** 853-854 (2012).
9. Eibauer, M. *et al.* Unraveling the structure of membrane proteins *in situ* by transfer function corrected cryo-electron tomography. *J. Struct. Biol.* **180,** 488-496 (2012).
10. Maimon, T., Elad, N., Dahan, I. & Medalia, O. The human nuclear pore complex as revealed by cryo-electron tomography. *Structure* **20,** 998-1006 (2012).
